# Significance of the Sparing Pronator Quadratus for Volar Plating of Distal Radius Fractures: A Prospective Study

**DOI:** 10.7759/cureus.74274

**Published:** 2024-11-22

**Authors:** Vishal S Champawat, Vaibhav Jain, Manish Rajpoot, Jai K Sahu, Deepak S Maravi

**Affiliations:** 1 Orthopaedics, Gandhi Medical College, Bhopal, Bhopal, IND

**Keywords:** distal radius fracture, distal radius plating, minimal invasive plate osteosynthesis distal radius, pronator quadratus, sparring pronator quadratus

## Abstract

Background

Pronator quadratus (PQ) acts as the pronator of the wrist and stabiliser of the distal radioulnar joint; it also provides a protective cover over the edge of the plate, preventing friction and subsequent rupture of flexor tendons. The repair of PQ is often difficult, and its durability is questionable; hence, preserving the PQ while volar plating distal radius fractures is advocated.

Methods

In this prospective randomised trial, 60 patients with a fracture of the distal end of the radius of AO-type (Arbeitsgemeinschaft für Osteosynthesefragen) 23 A2, A3, B1, B3, C1, and C2 were treated with volar plate fixation using either the PQ-releasing and repair approach (Group A, n = 30) or the PQ-sparing approach (Group B, n = 30), allowed by simple randomisation. Outcomes recorded included operative time, number of intraoperative radiation shots, range of motion, grip strength, Quick DASH (Disabilities of Arm, Shoulder, and Hand) score, visual analog scale (VAS) score, and complications, if any.

Results

Follow-up was done at 1, 3, 6, and 12 months. In the PQ-releasing group, the mean operative time was 54 ± 13 minutes, and the mean number of C-arm shots was 22 ± 7, whereas in the PQ-sparing group, the mean operative time was 82 ± 15 minutes, and the mean number of C-arm shots was 37 ± 7. Palmar flexion, supination, and pronation were significantly better at all the follow-ups until 12 months; however, dorsiflexion was better only until six months in the PQ-sparing group when compared to the PQ-releasing group. DASH score, VAS score, and grip strength were also significantly better until six months in the PQ-sparing group, with no significant difference at the 12-month follow-up in both groups.

Conclusions

There is statistically significant earlier functional recovery by preserving the PQ muscle for volar plating of distal radius fractures, with no increase in complication rate, and an attempt to preserve the PQ should be made.

## Introduction

The incidence of distal radius fractures is around 18% in the adult population [1]. Volar plate fixation is the treatment of choice for unstable distal radius fractures. The pronator quadratus (PQ) muscle has two heads: the superficial head, which is responsible for forearm pronation, and the deep head, which is responsible for the stabilisation of the distal radioulnar joint [2]. The PQ muscle may also provide a protective cover over the edge of the plate, preventing friction and subsequent rupture of the flexor tendons [3].

The PQ is supplied by a major pedicle from the anterior interosseous artery and minor pedicles from the radial and ulnar arteries. Furthermore, numerous branches from the PQ supply the metaphyseal and epiphyseal regions of the distal radius, and these branches may be important in the healing of distal radius fractures. Injury to them may increase the non-union rate [4-6]. The modified Henry approach requires an incision of the PQ from its radial attachment and its elevation to the sigmoid notch to expose the fracture; this may compromise the blood supply.

The muscle-to-muscle repair of the PQ in the modified Henry approach is difficult in high-energy trauma, in which the PQ is contused, friable, and often partially torn by displaced fracture fragments [7,8]. The resultant postoperative fibrosis of such an injured muscle, after repair, can lead to impaired forearm rotation [8]. The aim of this study was to investigate the clinical and functional outcomes of distal radius fractures fixed using the PQ-sparing approach and to compare them with those of the PQ-releasing approach.

We hypothesised that there would be a significant difference, with the PQ-sparing technique having a better functional outcome and a lower complication rate compared with the PQ-releasing approach.

## Materials and methods

After the Institutional Ethics Committee’s approval (approval number: 119/IEC/2022), a prospective study was conducted between August 2022 and September 2023. The study population was divided into two groups: Group A (PQ-releasing) and Group B (PQ-sparing) by means of simple randomisation. Inclusion criteria were age >18 years, AO classification 23 A2, A3, B1, B3, C1, and C2 (isolated and closed fractures). Exclusion criteria were open fractures; ipsilateral fresh or old upper limb fractures (including ulnar styloid fractures); fractures associated with neurovascular injury; cases with significant laceration found in PQ intraoperatively; and cases in Group B in whom repair of PQ could not be achieved. After the exclusion of cases and those lost to follow-up, the study was stopped when the study population consisted of 60 patients, with 30 patients in each group.

In Group A, the modified Henry approach was used. The PQ was exposed and incised with a blade knife (cautery was not used) along its distal and radial borders, leaving 2-3 mm of tissue. The PQ was then elevated with a periosteal elevator. After fixation, the PQ was repaired with figure-of-eight stitches at both released borders.

**Figure 1:**
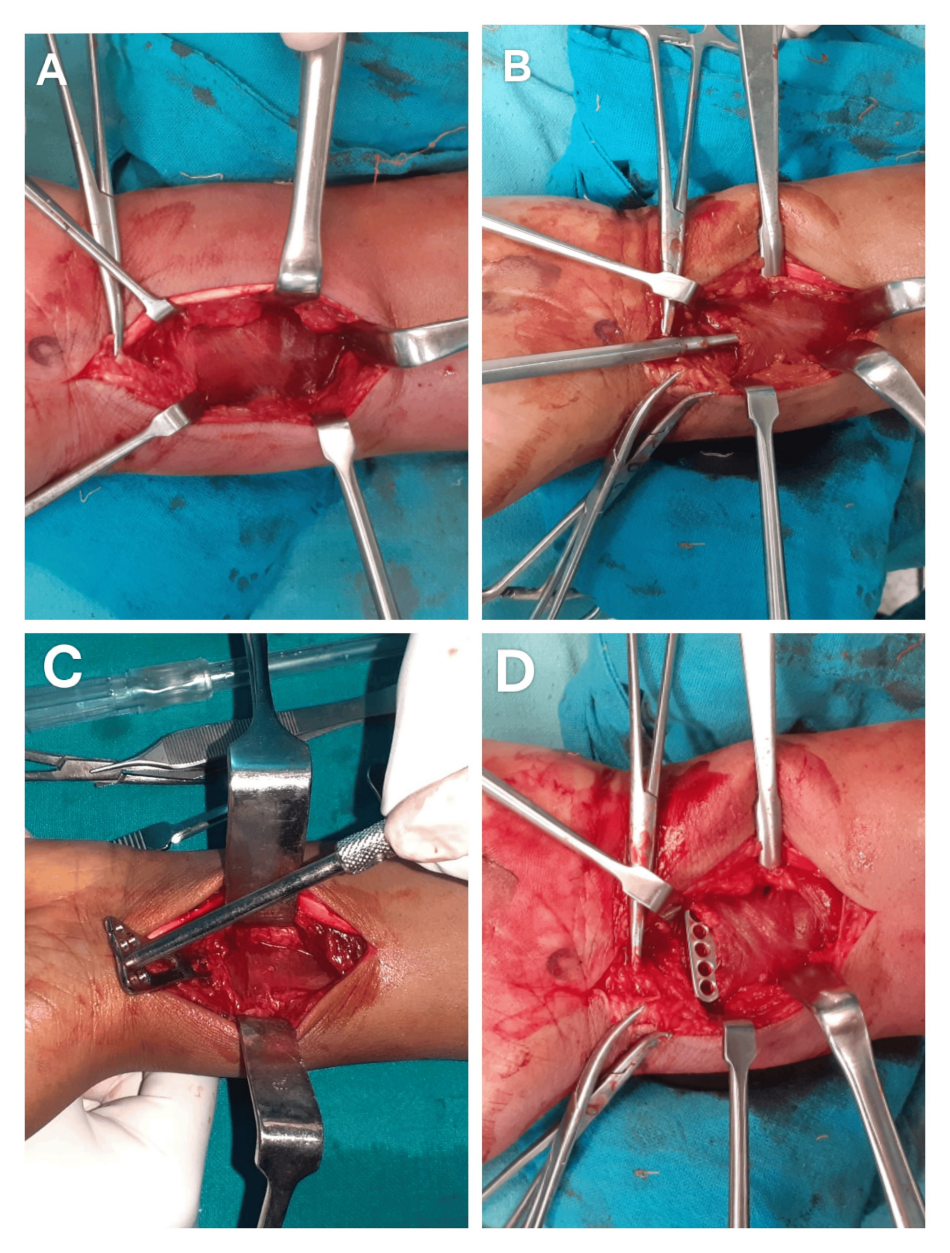
Steps of pronator quadratus sparing approach (A) Exposure of pronator quadratus; (B) Submuscular elevation of pronator quadratus; (C) Sliding of plate underneath pronator quadratus; (D) Final seating of plate prior to screw insertion

In Group B, the modified Henry approach was used. The PQ was exposed, and a transverse incision was made along its distal border, followed by retrograde elevation of the PQ to create a tunnel between the PQ and distal radius (Figure 1). Any remaining malreduction was corrected at this stage. An appropriately sized plate was then slid retrograde underneath the PQ, and its position was confirmed under fluoroscopy. The screw holes of the longitudinal limb of the plate were exposed using mosquito forceps, separating muscle fibers of the PQ along their length. The screw holes in the transverse limb were exposed by retracting the PQ proximally with a right-angle retractor.

In postoperative care, edema control measures and active finger range of motion (ROM) were initiated immediately. A short arm splint was applied for 14 days, after which sutures were removed and hand therapy was initiated. Follow-up was done at 1, 3, 6, and 12 months. The following variables were recorded: operative time, number of intraoperative radiation shots, ROM, grip strength, Quick DASH (Disabilities of Arm, Shoulder, and Hand) score, visual analog scale (VAS) score, and complications, if any.

Statistical analysis was done using Student’s unpaired t-test (parametric test), where the means of the two intervention groups were compared. To test the association between categorical variables, the Chi-square test (non-parametric test) was used. The level of significance, i.e., a p-value <0.05, was considered significant.

## Results

**Figure 2:**
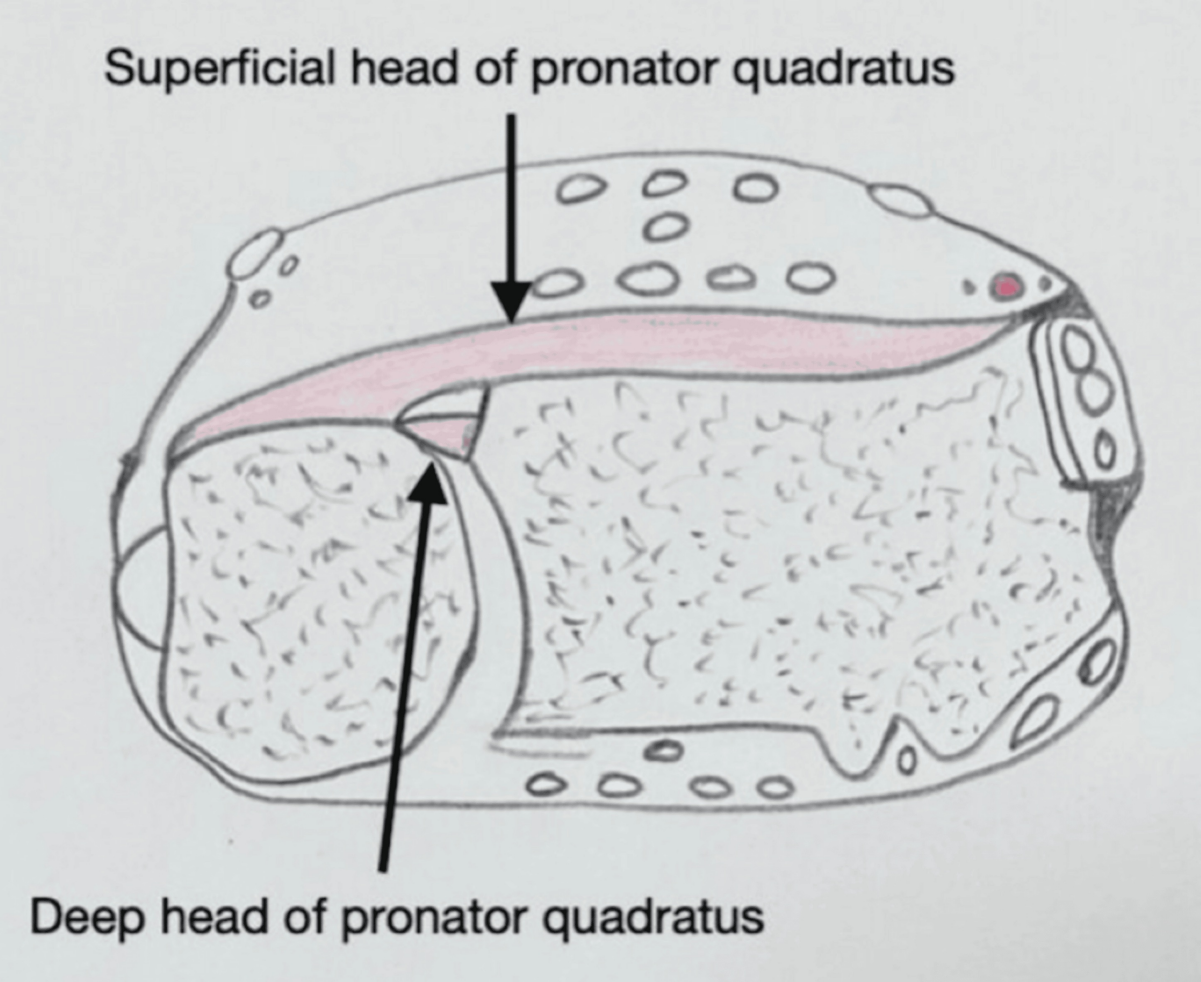
Line diagram illustrating PQ two heads Image credit: Dr. Jai K. Sahu PQ, Pronator quadratus

**Figure 3:**
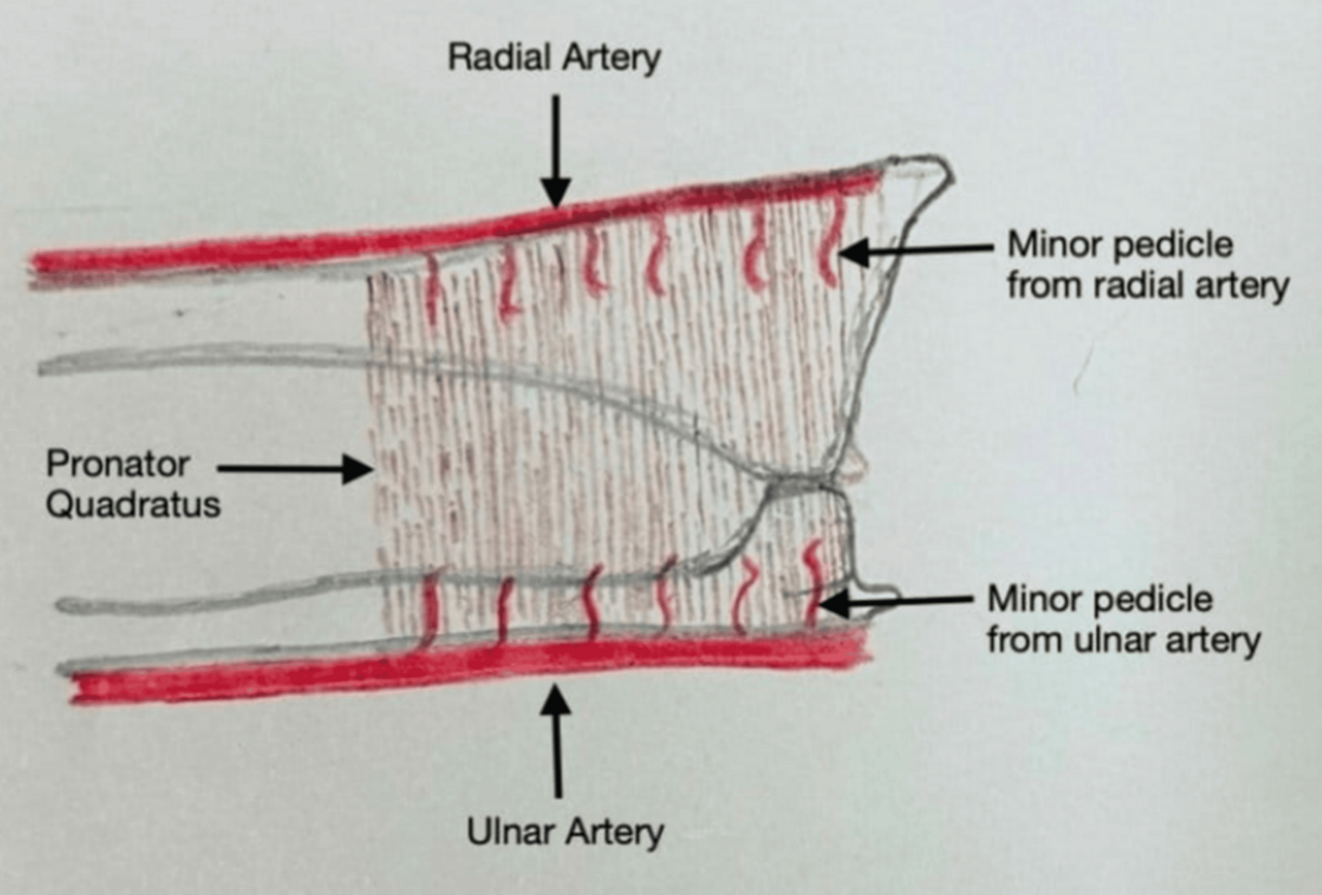
Minor pedicles from radial artery supplying pronator quadratus Image credit: Dr. Jai K. Sahu

Figure 2 depicts the two heads of the PQ, and Figure 3 depicts the minor pedicles arising from the radial artery, supplying the PQ.

The study population initially consisted of 78 patients, from which 18 were lost to follow-up, leaving 60 patients (30 in Group A and 30 in Group B) until the final follow-up. All patients underwent union of fractures. Group A consisted of 23 males/7 females and 16 right-sided/14 left-sided wrist fractures. Group B consisted of 19 males/11 females and 13 right-sided/17 left-sided wrist fractures (Table 1).

**Table 1:** Baseline characteristics of two patients groups

		Group A (pronator quadratus releasing approach)	Group B (pronator quadratus sparing approach)	Total
Mean age		39.4 ± 13.4	38.3 ± 14.9	38.5 ± 14.4
Gender	Male	23	19	42 (70%)
	Female	7	11	18 (30%)
Mode of injury	Fall on to outstretched hand	20	17	37 (61.7%)
	Road traffic accident	10	13	23 (38.3%)
Side of injury	Right	14	17	29 (48.3%)
	Left	16	13	31 (51.7%)

The mean operative time in Group A was 54 ± 13 minutes, and in Group B, it was 82 ± 15 minutes. The mean number of C-arm shots in Group A was 22 ± 7, and in Group B, it was 37 ± 7. Table 2 depicts the fracture distribution in the two groups according to the AO (Arbeitsgemeinschaft für Osteosynthesefragen) classification.

**Table 2:** Distribution of patient according to AO classification for both intervention groups AO, Arbeitsgemeinschaft für Osteosynthesefragen

AO classification	Open reduction and volar plating with pronator quadratus releasing approach (n = 30)		Open reduction and volar plating with pronator quadratus sparing approach (n = 30)		Total (N = 60)	
	No.	%	No.	%	No.	%
23A2	12	40	10	33.3	22	36.7
23B1	6	20	5	16.7	11	18.3
23B2	4	13.3	6	20	10	16.7
23C1	5	16.7	4	13.3	9	15
23C2	3	10	5	16.7	8	13.3
Total	30	100	30	100	60	100

Palmar flexion, supination, and pronation were significantly better at all the follow-ups until 12 months; however, dorsiflexion was better only until six months in Group B (sparing) when compared to Group A (releasing).

DASH score, VAS score, and grip strength were also significantly better only until six months in Group B (sparing) when compared to Group A (releasing), with no significant difference at the 12-month follow-up (Table 3). Complications occurred in the PQ-sparing group; one patient developed a surgical site infection, and in the PQ-releasing group, one patient developed a flexor pollicis longus rupture.

**Table 3:** Functional outcome of two groups PQ, Pronator quadratus; DASH, Disabilities of arm, shoulder, and hand; VAS, Visual analog scale

		6 weeks	3 months	6 months	12 months
Dorsi flexion	PQ-releasing	48.2 ± 2.94	58.3 ± 2.01	65 ± 1.84	68.6 ± 1.56
	PQ-sparing	53.23 ± 2.97	60.97 ± 3.77	67 ± 4.2	69.47 ± 3.39
	p-value	<0.00001	0.0011	0.037	0.20
Palmar flexion	PQ-releasing	53.0 ± 4.02	61.27 ± 3.23	69 ± 3.27	73.1 ± 1.43
	PQ-sparing	57.1 ± 3.48	64.9 ± 3.45	72 ± 2.65	74.0 ± 0.93
	p-value	<0.00009	0.00028	0.00029	0.004
Supination	PQ-releasing	63.4 ± 5.25	70.9 ± 4.12	76 ± 2.81	80.4 ± 1.83
	PQ-sparing	70.0 ± 4.27	76.9 ± 3.77	82 ± 3.11	83.9 ± 1.60
	p-value	<0.00001	<0.00001	<0.00001	<0.00001
Pronation	PQ-releasing	57.0 ± 1.50	64.9 ± 1.48	70 ± 1.71	73.27 ± 1.78
	PQ-sparing	60.8 ± 2.02	69.1 ± 1.51	72 ± 1.24	74.9 ± 0.55
	p-value	<0.00001	<0.00001	0.00001	<0.00001
Ulnar deviation	PQ-releasing	19.3 ± 2.75	23.2 ± 2.85	26.8 ± 2.96	30.27 ± 3.12
	PQ-sparing	19.9 ± 2.83	23.9 ± 3.09	27.1 ± 3.11	30.9 ± 3.36
	p-value	0.27	0.366	0.642	0.453
Radial deviation	PQ-releasing	13.8 ± 1.37	15.2 ± 1.4	16.5 ± 1.48	17.7 ± 1.53
	PQ-sparing	14.1 ± 1.53	15.4 ± 1.47	16.6 ± 1.44	17.9 ± 1.47
	p-value	0.42	0.593	0.725	0.495
Grip power	PQ-releasing	30.6 ± 0.88	33.9 ± 1.39	36.5 ± 1.47	39.5 ± 0.86
	PQ-sparing	32.4 ± 0.96	34.9 ± 0.99	37.5 ± 0.91	39.82 ± 0.94
	p-value	<0.00001	0.0021	0.0024	0.136
DASH score	PQ-releasing	19.97 ± 3.11	14.9 ± 3.43	10.3 ± 3.39	7.21 ± 3.2
	PQ-sparing	14.6 ± 3.08	11.1 ± 2.96	8.5 ± 2.8	5.7 ± 2.6
	p-value	<0.00001	<0.00002	0.028	0.06
VAS score	PQ-releasing	4.67 ± 1.15	3.27 ± 1.1	2.0 ± 1.1	0.83 ± 0.98
	PQ-sparing	3.6 ± 0.89	2.5 ± 0.78	1.4 ± 0.76	0.4 ± 0.7
	p-value	0.0002	0.002	0.012	0.07

**Figure 4:**
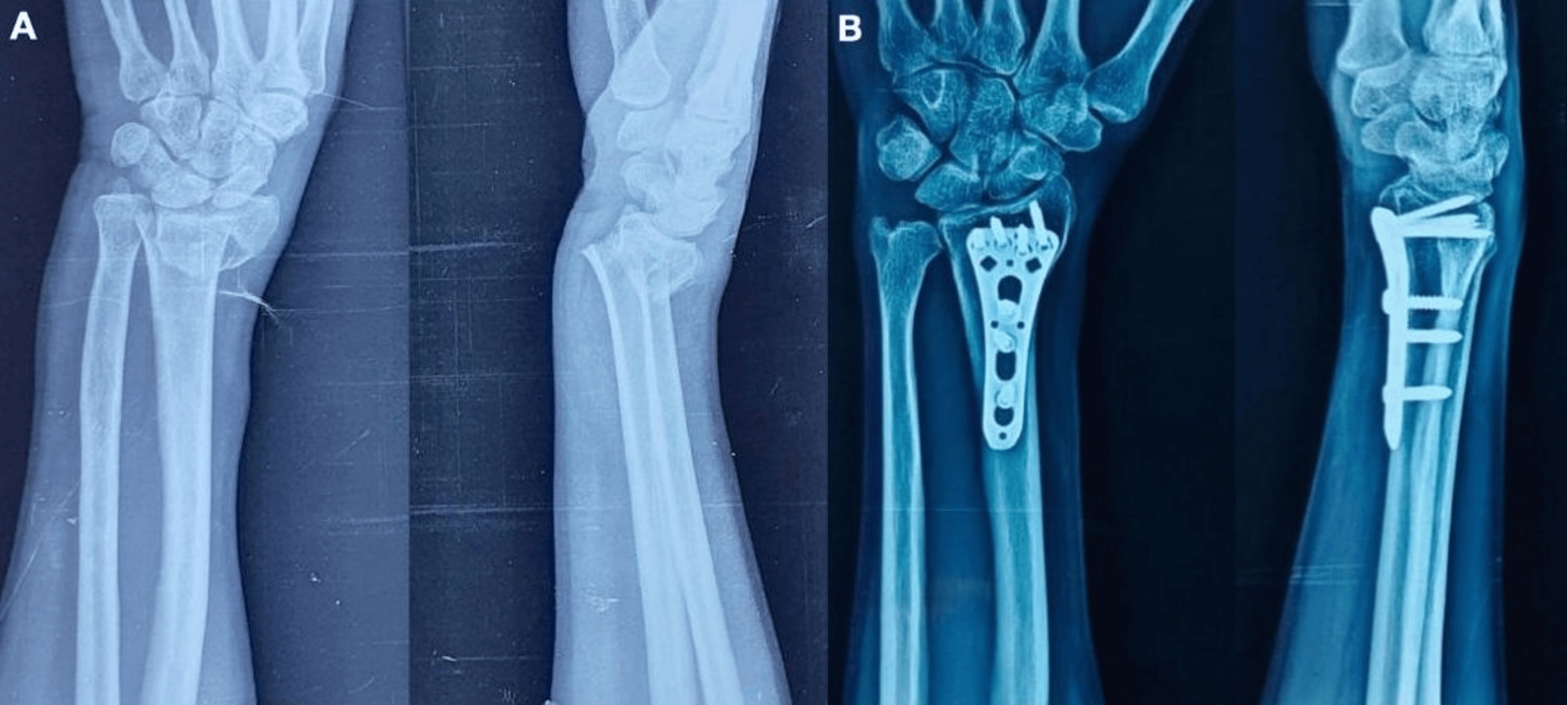
Radiological outcome of a case treated with pronator quadratus sparing approach (A) Preoperative X-ray; (B) 12 months follow-up X-ray

**Figure 5:**
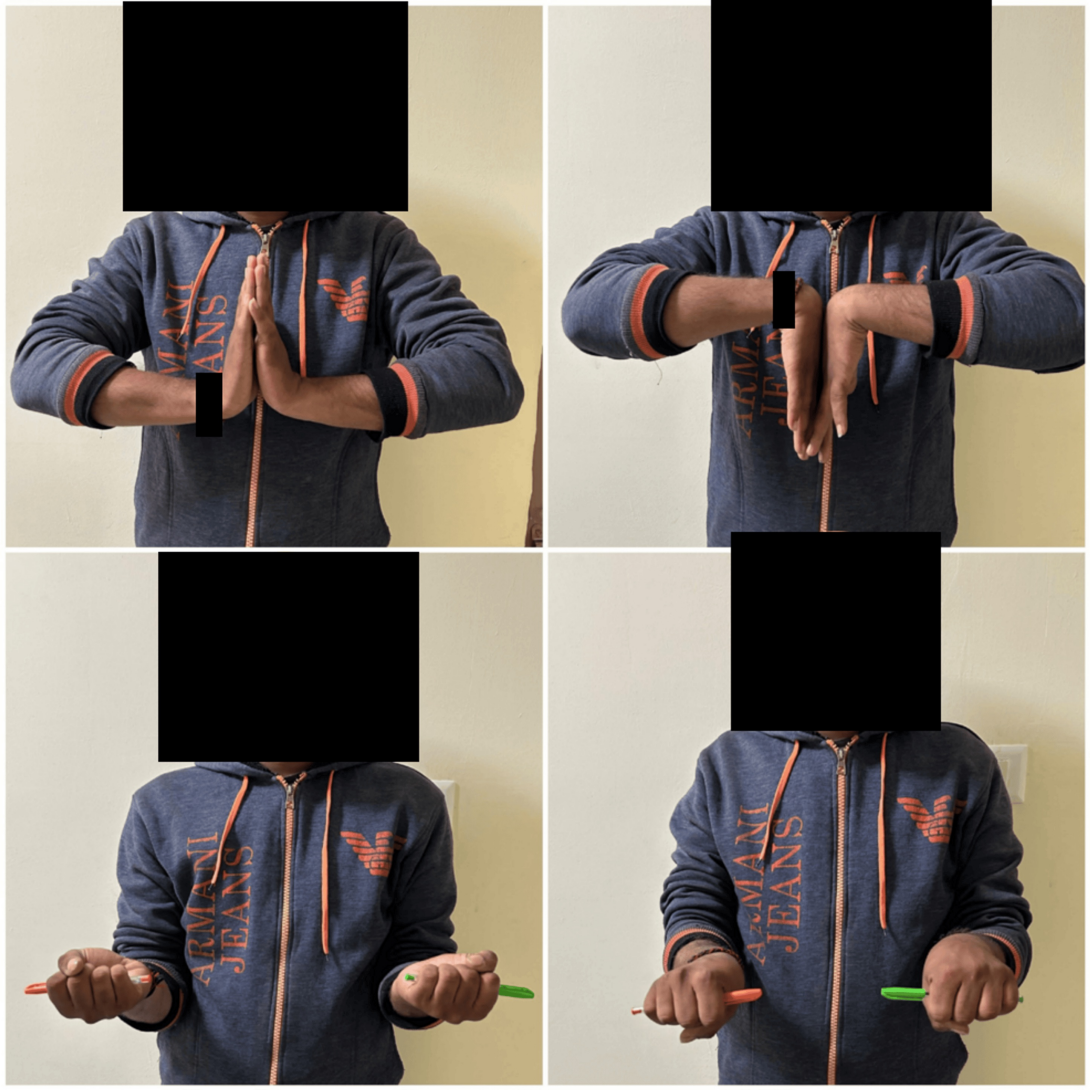
Clinical result at 12 months

Figures 4-5 depict the outcome of a patient treated with the PQ-sparing approach.

## Discussion

In this prospective randomised study, we compared the PQ-sparing technique with the PQ-releasing technique for distal radius volar plating. The mean operative time and mean number of C-arm shots were higher in the sparing approach group. Wrist palmar flexion, supination, and pronation were significantly better at all follow-ups; however, wrist dorsiflexion, VAS score, DASH score, and grip strength were better only up to six months in the sparing approach group, as compared to the releasing approach group. There was no difference in radial and ulnar deviation at any of the follow-ups in the two groups.

Previous studies have found varying results. Cannon et al. [9] found similar short-term radiological outcomes (radial height, radial inclination, and articular step-off) in the PQ-sparing and conventional approach groups, and both groups had similar tourniquet time (81 minutes). Itoh et al. [3] compared the two approaches up to 12 months postoperatively and found that the mean VAS score was superior until four months, and the Quick DASH score was superior until two months postoperatively in the PQ-sparing group compared with the PQ-releasing group. Meanwhile, there were no significant differences between the groups in any other functional parameters, including ROM and grip strength, throughout the observed period. Huang et al. [10] compared the two approaches and found that the mean operative time (p = 0.00), mean intraoperative blood loss (p = 0.00), and mean fracture union time (p = 0.034) were better in the sparing group. ROM and VAS scores were better for up to three months in the sparing group. No significant difference was found at 12 months in any of the parameters between the two groups. Three patients developed flexor tendon irritation, and two developed delayed carpal tunnel syndrome in the conventional approach group. Zhong et al. [11] performed PQ preservation in 41 patients, and all patients achieved satisfactory clinical results without any complications. Fourteen patients underwent hardware removal, and the postoperative muscle structures were found to be similar to the muscle structures before the first operation.

Sen et al. [7] were the first authors to describe this pronator-sparing surgical technique. The expected advantage of PQ-sparing over releasing and repairing is reduced bleeding, as the approach protects the minor vascular pedicles arising from the radial artery entering the PQ from being sacrificed. Also, numerous branches from the PQ supply the metaphyseal and epiphyseal regions of the distal radius, and preserving these branches may lead to early fracture healing [4,6]. Preservation of pronation strength and stability of the distal radioulnar joint [3] is important, as the superficial head of the PQ is responsible for forearm pronation, while the deep head is responsible for stabilising the distal radioulnar joint [2]. PQ contributes to 21% of pronation torque [12], and Armangil et al. [13] demonstrated a mean loss of 18.5% in pronation strength after repairing the PQ for plating distal radius fractures, with a minimum of six months follow-up. An incised muscle heals with a connective tissue scar [14], and this scarred muscle can lead to restriction in the rotation of the forearm.

**Figure 6:**
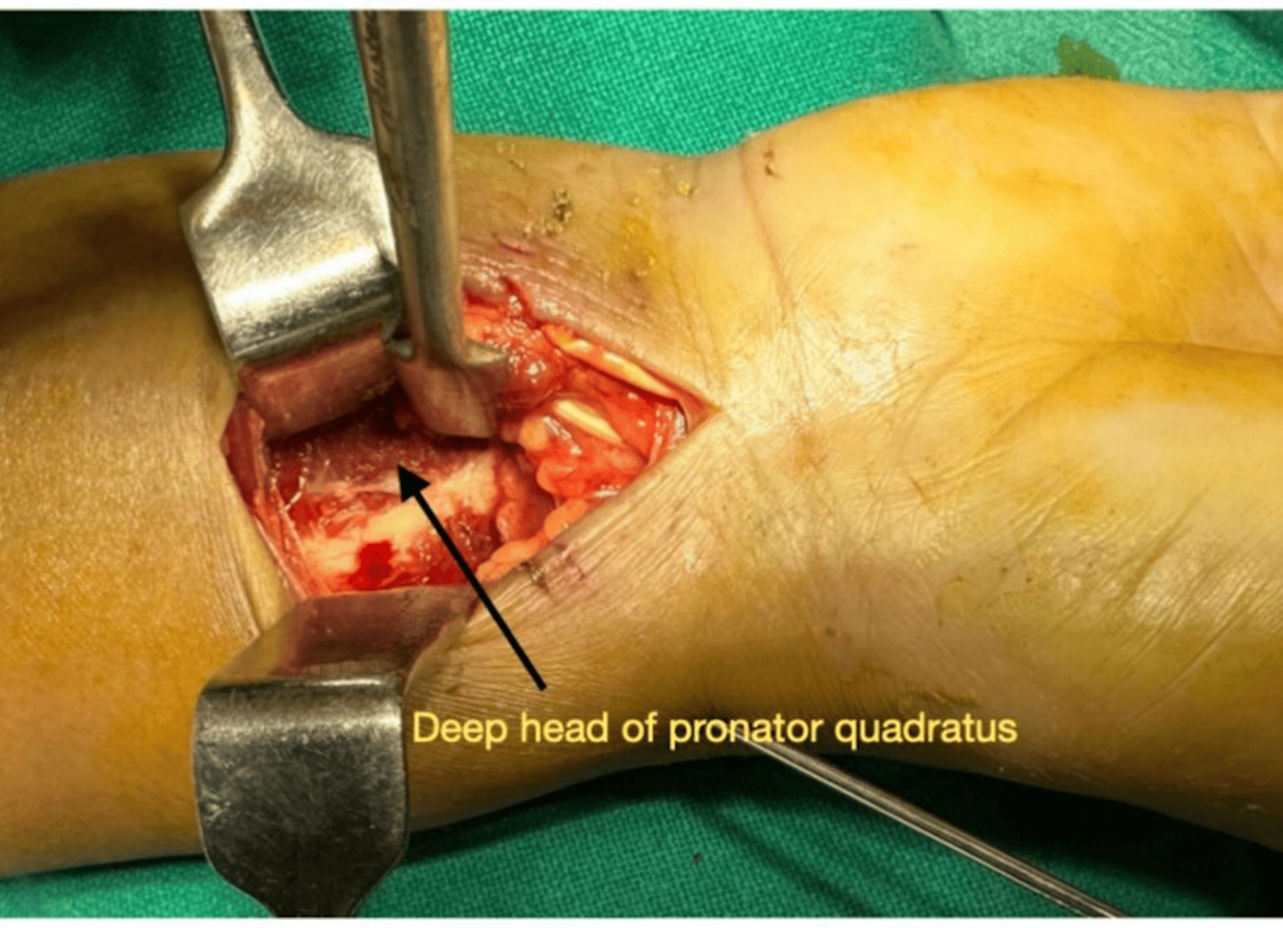
Exposure of deep head of the pronator quadratus

The reason for no significant statistical difference in various parameters at the final follow-up in our study and other studies may be because: (I) the deep head of the PQ (stabiliser of the distal radioulnar joint) remains intact (Figure 6) even after the PQ-releasing approach, as described by Sonntag et al. [15], who performed an ultrasound evaluation of the PQ following volar plating with the conventional PQ-releasing approach. They found that the deep head of the PQ muscle was intact with normal morphology in all participants. (II) The repair of the PQ is durable, as described by Sonntag et al. [15], who assessed the PQ repair and found it intact in all patients at three months in whom it was attempted. Hinds et al. [16] assessed PQ repair integrity after repairing the PQ for plating distal radius fractures, at a minimum of three months post-op in 20 patients, and found that all patients had an intact PQ repair. Swigart et al. [17] reported that repair of the PQ muscle is generally durable, with a 4% failure rate three months after surgery. The recovery of pronation torque with strengthening exercises and rehabilitation may be near normal, as described by Armangil et al. [13]. They compared pronation torque following volar plating of distal radius fractures and PQ repair with their uninjured wrist and documented that two patients, who had further strengthening exercises beyond their own rehabilitation regimes, had better pronator torque compared to their uninjured forearm.

A few of the limitations of this study are its small sample size, short duration of follow-up, and risk of bias due to the simple randomisation used for group allotment to patients. A future multicenter study, consisting of a large sample size with long-term follow-up, would be beneficial.

## Conclusions

In this study, statistically significant early functional recovery was achieved by preserving the PQ muscle for volar plating of distal radius fractures, with a similar complication rate to the PQ-releasing approach. We highly recommend the preservation of PQ for volar plating of distal radius fractures. However, fracture reduction should be given paramount importance, rather than the surgical approach, and the PQ-preserving approach should be converted into the PQ-releasing approach if any difficulty is encountered in fracture reduction during surgery.

## References

[1] Nellans KW, Kowalski E, Chung KC (2012). The epidemiology of distal radius fractures. Hand Clin.

[2] Johnson RK, Shrewsbury MM (1976). The pronator quadratus in motions and in stabilization of the radius and ulna at the distal radioulnar joint. J Hand Surg Am.

[3] Itoh S, Yumoto M, Kanai M, Yoshida W, Yoshioka T (2016). Significance of a pronator quadratus-sparing approach for volar locking plate fixation of comminuted intra-articular fractures of the distal radius. Hand (NY).

[4] Lamas C, Llusà M, Méndez A, Proubasta I, Carrera A, Forcada P (2009). Intraosseous vascularity of the distal radius: anatomy and clinical implications in distal radius fractures. Hand (NY).

[5] Thomas BP, Kiran SP, Tang M, Geddes CR, Morris SF (2021). The vascular basis of the pronator quadratus muscle flap and its use in clinical cases. Indian J Plast Surg.

[6] Rath S, Hung LK, Leung PC (1990). Vascular anatomy of the pronator quadratus muscle-bone flap: a justification for its use with a distally based blood supply. J Hand Surg Am.

[7] Sen MK, Strauss N, Harvey EJ (2008). Minimally invasive plate osteosynthesis of distal radius fractures using a pronator sparing approach. Tech Hand Up Extrem Surg.

[8] Heidari N, Clement H, Kosuge D, Grechenig W, Tesch NP, Weinberg AM (2012). Is sparing the pronator quadratus muscle possible in volar plating of the distal radius?. J Hand Surg Eur Vol.

[9] Cannon TA, Carlston CV, Stevanovic MV, Ghiassi AD (2014). Pronator-sparing technique for volar plating of distal radius fractures. J Hand Surg Am.

[10] Huang X, Jia Q, Li H (2022). Evaluation of sparing the pronator quadratus for volar plating of distal radius fractures: a retrospective clinical study. BMC Musculoskelet Disord.

[11] Zhong J, Li X, Lv F, Cao N, Li D (2022). Clinical applications of internal fixation via the volar approach with pronator quadratus preservation for distal radius fractures. Turk J Med Sci.

[12] McConkey MO, Schwab TD, Travlos A, Oxland TR, Goetz T (2009). Quantification of pronator quadratus contribution to isometric pronation torque of the forearm. J Hand Surg Am.

[13] Armangil M, Bezirgan U, Başarır K, Bilen G, Demirtaş M, Bilgin SS (2014). The pronator quadratus muscle after plating of distal radius fractures: is the muscle still working?. Eur J Orthop Surg Traumatol.

[14] Lehto MU, Järvinen MJ (1991). Muscle injuries, their healing process and treatment. Ann Chir Gynaecol.

[15] Sonntag J, Hern J, Woythal L, Branner U, Lange KH, Brorson S (2021). The pronator quadratus muscle after volar plating: ultrasound evaluation of anatomical changes correlated to patient-reported clinical outcome. Hand (NY).

[16] Hinds RM, Montero-Lopez N, Brock K, Adler R, Sapienza A, Capo JT, Paksima N (2020). Assessment of pronator quadratus repair integrity using dynamic ultrasonography following volar plate fixation for distal radius fractures. Hand (NY).

[17] Swigart CR, Badon MA, Bruegel VL, Dodds SD (2012). Assessment of pronator quadratus repair integrity following volar plate fixation for distal radius fractures: a prospective clinical cohort study. J Hand Surg Am.

